# Policy implementation in wheelchair service delivery in a rural South African setting

**DOI:** 10.4102/ajod.v2i1.63

**Published:** 2013-09-09

**Authors:** Surona Visagie, Elsje Scheffler, Marguerite Schneider

**Affiliations:** 1 Centre for Rehabilitation Studies, Stellenbosch University, South Africa; 2 DARE Consult, Stellenbosch, South Africa; 3 Department of Psychiatry and Mental Health, University of Cape Town, South Africa

## Abstract

**Background:**

Wheelchairs allow users to realise basic human rights and improved quality of life. South African and international documents guide rehabilitation service delivery and thus the provision of wheelchairs. Evidence indicates that rehabilitation policy implementation gaps exist in rural South Africa.

**Objectives:**

The aim of this article was to explore the extent to which wheelchair service delivery in a rural, remote area of South Africa was aligned with the South African National Guidelines on Provision of Assistive Devices, The United Nations Convention on the Rights of Persons with Disabilities and The World Health Organization Guidelines on Provision of Wheelchairs in Less-Resourced Settings.

**Method:**

Qualitative methods were used. Data were collected through semi-structured interviews with 22 participants who were identified through purposive sampling. Content analysis of data was preformed around the construct of wheelchair service delivery.

**Results:**

Study findings identified gaps between the guiding documents and wheelchair service delivery. Areas where gaps were identified included service aspects such as referral, assessment, prescription, user and provider training, follow up, maintenance and repair as well as management aspects such as staff support, budget and monitoring. Positive findings related to individual assessments, enthusiastic and caring staff and the provision of wheelchairs at no cost.

**Conclusion:**

The gaps in policy implementation can have a negative impact on users and the service provider. Inappropriate or no wheelchairs limit user function, participation and quality of life. In addition, an inappropriate wheelchair will have a shorter lifespan, requiring frequent repairs and replacements with cost implications for the service provider.

## Introduction

The provision of assistive devices to compensate for loss of function is an essential part of rehabilitation (United Nations [UN] 2006a). A wheelchair, defined as ‘a device providing wheeled mobility and seating support for a person with difficulty in walking or moving around’, is one such assistive device (World Health Organization [WHO] 2008:11).

Functionality in a wheelchair relies on the interaction between the user, the wheelchair, the environment and the activity performed (Routhier *et al.* 2003). Wheelchair provision in rural areas must, therefore, take into account the uneven terrain, lack of paved roads and sidewalks, eroded paths, small houses and narrow doors often found in these settings (Eldar 2001). In addition, transport services are often limited in low-resourced settings and wheelchairs become the primary mode of mobility (Schoenberg & Coward 1998; WHO 2008). The WHO takes into consideration such contextual variables and defines an appropriate wheelchair as one that:

[*M*]eets the user’s needs and environmental conditions; provides proper fit and postural support; is safe and durable, and can be obtained and maintained and services sustained in the country at the most economical and affordable price. (WHO 2008:11)

An appropriate wheelchair plays a key role in enhancing quality of life of the user as it can assist with the realisation of basic human rights such as access to health care and education, economic self-sufficiency and participation in community and social life (Borg, Lindstrom & Larsson 2009; Department of Health [DoH] 2003; Greer, Brasure & Wilt 2012; UN 2006b; WHO 2008). Furthermore, literature associates a correctly-prescribed wheelchair with improved function and healthcare benefits through the prevention of secondary complications (DoH 2003; UN 2006b). Provision of appropriate wheelchairs should thus be one of the priorities of a rehabilitation programme.

The aim of this article is to explore the extent to which wheelchair service delivery in the study setting is aligned with National Policy, The United Nations Convention on the Rights of Persons with Disabilities (UNCRPD) (Articles 4, 20 and 26) (UN 2006b) and the WHO Guidelines on Provision of Wheelchairs in Less-Resourced Settings (WHO Guidelines) (WHO 2008). The experiences of public-sector healthcare users and service providers with regards to wheelchair services in a remote, rural district in the Northern Cape Province of South Africa are described, as 80% of the South African population relies on government subsidised healthcare services (Blecher *et al*. 2011). These experiences were then used to further determine if there were gaps in policy implementation.

Whereas the limited provision of rehabilitation services in remote, rural settings is generally acknowledged (Bateman 2012), analysis and documentation of the gap between policy and implementation in specific areas of service provision is lacking and this article aims to add to that evidence base.

### Local and international policy

The South African (SA) National Rehabilitation Policy (NRP) (DoH 2000) guides general rehabilitation services in South Africa, whilst the South African National Guidelines on Provision of Assistive Devices (SA National Guidelines) stipulate key responsibilities and requirements with regard to the provision of assistive devices in the public-health sector in South Africa (DoH 2003). Both of these documents promote a user-centric approach and highlight important programme-management elements such as training of staff, budgeting and monitoring and evaluation. In addition, the SA National Guidelines include specific protocols with regard to key service steps in the provision of wheelchairs and other assistive devices.

The UNCRPD (UN 2006b) promotes a rights-based approach to service delivery. With reference to assistive technology, Article 4 seconds the WHO definition of an appropriate wheelchair by promoting the development of appropriate devices and technology, training of staff and the dissemination of information on assistive technology to users. Article 20 promotes personal mobility through service delivery, provision of quality devices and user training. Rehabilitation service-management aspects are covered in Article 26 and the need for dedicated rehabilitation programmes, capacity building of staff and provision of assistive devices are outlined (UN 2006b).

The WHO guidelines (WHO 2008) outline eight service steps that form the basis of a comprehensive wheelchair service. In addition, the WHO guidelines include minimum standards for each step. These service steps and minimum standards are based on international evidence-based practice and research (Greer *et al*. 2012; WHO 2008). The service steps are: (1) referral and appointment; (2) assessment; (3) prescription; (4) funding and ordering; (5) product preparation; (6) fitting; (7) user training; and (8) follow up, maintenance and repairs. (As ‘follow up’ includes multiple factors in addition to technical aspects, it will be discussed separately [a] from ‘maintenance and repairs’ [b]).

The SA National Guidelines (DoH 2003) include most of the service steps outlined in the WHO Guidelines (WHO 2008), but there is limited clinical focus and scope and there are limited standards for each service step. For example, the main clinical focus in the service steps relating to assessment, prescription and fitting is that they must be performed by appropriately-trained staff. No specific clinical requirements are given. The following service steps are not included in the SA National Guidelines (DoH 2003):

- Referral and appointment.
- Product preparation.
- Follow up.

Despite the differences between the documents, the SA National Guidelines can, in principle, be aligned with the WHO Guidelines.

### Policy implementation

According to Lang *et al*. (2011), the successful implementation of international policy in individual countries depends on country-level policy that directs implementation, political will and adequate government structures to facilitate implementation. South Africa has country-level policy in the form of the NRP (DoH 2000) and the SA National Guidelines (DoH 2003), which predate both the UNCRPD and the WHO Guidelines. In addition, South Africa ratified and signed the UNCRPD in 2007, thus voluntarily committing itself to act on the articles of the Convention. Together, these policies and the ratification of the UNCRPD should provide a sound foundation for the implementation of the international documents. However, it is documented that rehabilitation service provision is inadequate in rural South Africa despite the presence of these policies and guidelines (Bateman 2012). What is unclear is to what extent local and international policy are implemented with regard to wheelchair service delivery in rural South Africa.

## Research method and design

Information presented in this article was gathered during a larger study entitled ‘Enabling universal and equitable access to health care for vulnerable people in resource poor settings’ (EquitAble 2008). The EquitAble study explored access to healthcare by people seen as vulnerable in four sites in each of four African countries. South Africa was one of those countries and, within South Africa, Fraserburg, the site on which this article is based, was one of the sites. The interviews conducted for the article formed part of the qualitative phase of the EquitAble project. A descriptive, inductive design was used to gain an in-depth understanding of the situation with regards to healthcare access in the study community (EquitAble 2008).

Data were collected in the town of Fraserburg, a geographic service area (GSA) in the Northern Cape Province of South Africa. Fraserburg and the Northern Cape Province have ‘limited financial, human and infrastructural resources to provide wheelchairs’ (WHO 2008:2). The GSA spans 10 000 km^2^ and is sparsely populated (an average of three persons per km^2^). This area has an expanded unemployment rate of 40% – 60%, including unemployed persons who are not looking for employment (Gaffneys Local Government in South Africa 2008). The transport infrastructure is poor with mostly gravel roads, footpaths and no public transport. In the low-income part of town, housing is most often a two- or three-roomed dwelling with narrow doors and stairs, unpaved yards, often with loose gravel surfaces and no access roads or paths. Healthcare services are nurse-driven and are provided at a Community Health Care Centre (CHCC). Doctors and therapists from the nearest secondary hospital, 200 km away, provide outreach services at the CHCC once a week.

A heterogeneous group of 22 study participants was recruited purposively from the Fraserburg general population and healthcare service providers in the GSA. Purposive sampling was used in order to ensure that participants who could shed the most light on healthcare access were interviewed (Domholdt 2005). Furthermore, participants were chosen so as to represent a heterogenic group with maximum variability in characteristics that might lead to different experiences and views on healthcare access (Domholdt 2005). The primary author, who is a member of the community and has experience in the field of physical rehabilitation, used her knowledge of the community to identify possible participants. In addition, political and religious leaders in the community were approached for names of healthcare users. Participants included eight healthcare users (both men and women) with ages ranging from 4 to 62 years (in the instance of children, legal guardians were interviewed); eight healthcare service providers whose duties included wheelchair service delivery (both providers based at the Fraserburg CHCC as well as those providing outreach services to the CHCC); and seven key informants who were members of the community and had a specific knowledge of healthcare service provision in the community. Of these participants, 10 either used or needed a wheelchair, or were involved in the management, prescription and procurement of wheelchairs.

Data were obtained through observations at the CHCC and from participants through semi-structured interviews, undertaken in three rounds by the primary author. After the initial round of 16 interviews in April 2010, the data were analysed provisionally and key issues were identified for further exploration. These included the procurement and provision of wheelchairs. A further five individuals who were knowledgeable on wheelchair services were identified and interviewed in June 2010. Through the analysis of these five interviews, it was decided to add a final interview with the person in charge of wheelchair provision in the Northern Cape in order to provide a perspective from the provincial level. In instances where initial analysis indicated a need for further exploration, the primary author went back to the specific participants for clarification.

All interviews were recorded digitally and then transcribed, after which the content was analysed by the primary author. The Framework approach to data analysis was used (Pope, Ziebland & Mays 2000).

Predetermined codes derived from the research question were used, but at the same time data were used to generate related and new themes. The themes were organised under the eight service steps described by the WHO guidelines (WHO 2008). Aspects considered in the initial coding included the words used, the context in which things were said, the frequency, extensiveness and intensiveness of comments, as well as internal consistency and specificity (Rabiee 2004). Narrative examples were extracted in order to illustrate the discussion of the findings. The qualitative analysis was conducted manually, that is to say, not by use of a software programme.

## Results

The results are presented with reference to the framework of the eight service steps of a comprehensive wheelchair service as stipulated by the WHO (WHO 2008). This facilitates the comparison of the ideal WHO Guidelines with what actually happens ‘on the ground’.

### Service step 1: Referral and appointment

According to the WHO Guidelines (WHO 2008), a clear referral network allows community sources and healthcare service providers to identify and refer persons who may benefit from wheelchairs. Although the NRP (DoH 2000) advocates the importance of referral pathways, no dedicated wheelchair referral system could be identified in the study setting. Wheelchair referrals followed the same referral path as general rehabilitation needs. It was found that only persons with a better prognosis were referred for wheelchairs, whereas those with poorer prognoses or lower levels of functioning were not referred. By doing this, wheelchair service providers did not follow a rights-based approach as independent function appeared to be prioritised over, for example, enhancing quality of life, reducing the burden of care, or restoring dignity. This was illustrated by a service provider who recognised the need for a wheelchair for a 4 year old child with physical and cognitive impairments, but failed to refer the child based on her poor level of functioning, leaving the child to crawl or be carried by the carer. In another example, a participant who suffered pelvic and hip fractures secondary to cancer was also not referred for a wheelchair:

‘We had to provide for ourselves. The wheelchair we got from a friend of mine … we bought the wheelchair from her.’ (P1, User, female, 63 years)

### Service step 2: Assessment

Assessment is the second service step of a comprehensive wheelchair service programme. The majority of good practice guidelines promote detailed individual assessments by trained staff and documentation on dedicated service forms (Health Professions Council of South Africa [HPCSA] 2008; Whitcombe-Shingler 2004; WHO 2008).

Although users were assessed individually and findings were documented on dedicated forms, assessments were incomplete and limited to personal income, body measurements and some basic accessibility and environmental factors:

‘I complete a form … they ask [*on the form*] income level … you have to measure the thigh length and the knee to the foot and the hip width … then it asks if there are steps and what the area around the house looks like.’ (P20, Provider, female, 24 years)

### Service step 3: Prescription

During prescription, the most appropriate wheelchair is selected by matching the technical specifications of the wheelchair with the user’s function, environmental, health and postural support needs (Moody *et al*. 2012; Whitcombe-Shingler 2004; WHO 2008), as determined by the assessment. Best practice and a rights-based approach advocate for education of, consultation with and active participation of the user (Borg 2011; Di Marco, Russell & Masters 2003; UN 2006b; WHO 2008), as well as for different wheelchair options to be demonstrated to the user. The user, where possible, should try out various wheelchair options in order to make an informed choice (Fogelberg *et al*. 2009; WHO 2008).

An interview with the person in charge of procurement in the study setting confirmed that no wheelchairs were available at the service centres for assessment and prescription purposes and that only the basic folding-frame wheelchair (orthopaedic hospital-style wheelchair) was available to be issued to users.

Toward the end of the study period the officer in charge of wheelchair distribution reported positive changes. These included improvements of the assessment form and prescribing appropriate wheelchairs from the full range on tender. However, ground-level service providers did not agree that these changes were implemented in the study setting.

### Service step 4: Funding and ordering

In Fraserburg, approval and authorisation of the prescribed wheelchair is carried out by a third party in the provincial procurement offices 800 km away. The distances alone cause inevitable delays in the supply of wheelchairs, with delays of up to four years reported. These delays can result in a loss of function and an inability to participate in family, community and economic life, and, for some, the indignity of spending their last days without any mobility:

‘[*W*]e have orders from 2006 that have not been delivered yet. The previous week we finally received a wheelchair. When I went to deliver the chair the woman had died.’ (P20, Provider, female, 24 years)

Services for children should be prioritised as they are a high-risk target group for rehabilitation interventions (DoH 2000, 2003; UN 2006b; WHO 2008). Parents of children using buggies (children-specific postural-support devices) reported significant improvement with regard to posture, mobility and function (Rigby, Ryan & Campbell 2009). However, they were also subject to delays:

‘[*I*]f the child is three years old and requires a buggy and the buggy is delivered in two years’ time then the child is five and the buggy will not fit anymore. It is a waste of money if you think about it.’ (P20, Provider, female, 24 years)

This statement also reflects a wastefulness of resources (the cost of a buggy is approximately $750 and is one of the more expensive devices available) (South African National Treasury 2010). The delay in provision is also potentially detrimental to the child’s functioning and health.

Budgetary constraints and unpredictable annual budgets were given as being the main reason for the long waiting lists and delayed provision. Driven by the need for mobility, any donated wheelchair, irrespective of type, size or user-specific needs, was accepted: ‘It depends on what APD [*Association for Persons with Physical Disabilities*] can get’ (P14, Key informant, male, 40 years).

At the time of the study, the financial shortfall was addressed through transferring funds from the hearing-aid provision programme, which was not fully implemented due to a lack of the relevant staff capacity:

‘[*L*]ast year the hearing aid budget was shared with us because there were too many hearing aids and too few therapists to fit hearing aids. So they have got a backlog of fitting instead of a backlog of waiting. So we got that part of the budget to clear our backlog and then next year we will split the budget again.’ (P24, Provider, female, age unknown)

Article 4 of the UNCRPD (UN 2006b) promotes free or subsidised provision of assistive devices. In accordance with this, the South African Uniform Patient Fee System (DoH 2009) allows for both free and subsidised provision of assistive devices based on a means test. Participants in receipt of social grants who took part in this study confirmed that they received their wheelchairs for free:

‘[*T*]hey give it. You just have to make sure that when you die, your family gives it back. So every time you get a new chair you give the other one back and when you die you give everything to the hospital.’ (P2, User, male, 63 years)

The wheelchair budget was managed from provincial level:

‘[*T*]here is a provincial budget. It has not been decentralised because therapists change so often in the district. There are no constant persons that can do the ordering from that level, so we have made it a provincial function.’ (P24, Provider, female, age unknown)

### Service step 5: Product preparation

This essential step (WHO 2008) ensures that the wheelchair matches the assessment and prescription findings and that it is safe and mechanically sound. As wheelchairs are not custom-fabricated for each user, each wheelchair requires individualised adjustment and/or customising in order to ensure that the configuration supports the optimal functional, postural support and health needs of the users. This step is also not listed as being a required service step in the SA National Guidelines, nor was it mentioned in any of the interviews.

### Service step 6: Fitting

Fitting aims to ‘ensure that the wheelchair fits correctly and supports the user as intended’ (WHO 2008:83). The SA National Guidelines (DoH 2003) fail to provide the scope for a proper fitting process and the results of this study suggested that this step was carried out superficially: ‘I set it up as I was taught at University, basically the footrests, give the cushion, put the brakes on’ (P20, Provider, female, 24 years).

### Service step 7: User training

Article 20(c) of the UNCRPD, the WHO guidelines and the SA National guidelines emphasise training of the user by trained service providers in mobility skills, general health care and basic device maintenance (DoH 2003; UN 2006b; WHO 2008.) Information from both service providers and users indicated a gap between guidelines and practice with regard to user training in Fraserburg. One of the service providers said: ‘I show him to reverse and things like that. Not very higher grade …’ (P20, Provider, female, 24 years)

It seems as if neither the service providers nor the users understood the risks involved in incorrect fitting or adjustments of the wheelchair. One participant-user reported:

‘Yes, the occupational therapist comes to see that these [*foot plates*] are tight and they give you a key in case the nuts and bolts get loose. They adjust the height for your legs [*footrests*]. If you want it higher or lower, you can set it yourself …’ (P2, User, male, 63 years)

When asked if he was trained in basic transfers and mobility skills, one user responded:

‘No. All they said is that I must exercise my arms a lot. Oh sorry, they also said I must try to walk. So my wife helps me here at home – she helps me get onto the bed and from the bed into the wheelchair.’ (P2, User, male, 36 years)

### Service step 8

#### Follow up

Follow up appointments are essential with regard to monitoring the on going appropriateness of the wheelchair fit, postural support, function and use in the environment (DoH 2003; Fogelberg *et al*. 2009; Hansen, Tresse & Gunnarson 2004; WHO 2008). As people’s health, activity level and environment may change over time, a different type of wheelchair or adjustments to the current one may be needed (Di Marco *et al*. 2003; Scherer 1996). Study findings indicated a lack of follow-up services and the SA National Guidelines (DoH 2003) also do not list follow up as being a service step. At the time of the study, one participant, whose wheelchair may have been a correct fit at the time of issue, was using a wheelchair which was too wide.

#### Maintenance and repairs

Despite evidence that routine inspection of wheelchairs decreases accidents, Hansen *et al*. (2004) found that users often do not report wheelchair deterioration in good time and therefore recommend regular mechanical maintenance in the user’s home every 12–24 months. According to the SA National Guidelines (DoH 2003), basic repairs should be preformed on a ‘fix-while-you-wait’ basis or within three days. Participants reported that there is only one official wheelchair repair centre, 800 km away from the study site: ‘[*T*]o fix it … they send it away … Then you wait; and it could take up to a year’ (P2, User, male, 63 years of age).

This was confirmed by the government official in charge of wheelchair services:

‘We have only got one [*repair centre*] in Kimberley, and none anywhere else in the province. So we have done workshops with the therapists, so some of them can do basic maintenance themselves, but the major overhaul and stuff needs to come to Kimberley. That is a challenge because then the client is without a chair … Sometimes what happens is that the patient comes to Kimberley with the chair and then they are here for the day and then the guys do the overhaul for the whole day and when the patient goes back, they go back with their chair. That is the ideal, but in cases where the maintenance cannot be done that fast, then they either get a loan chair or they get left without a chair.’ (P24, Provider, female, age unknown)

A positive finding was the pro-active approach to wait-listing of existing wheelchair users whose wheelchairs are reaching the end of their lifespan:

‘Look, a client who requires a second chair, they come in and they are reassessed and are put back onto the waiting list, depending on the condition of the chair. If the chair cannot be repaired, or if the last repair can now be done, and nothing after that. We already place the person back on the waitlist [*sic*].’ (P24, Provider, female, age unknown)

Although wheelchairs available on the tender have to pass stringent durability tests (South African National Treasury 2010), the durability requirement is also in line with the intended use of the chair. Despite adhering to durability specifications, the basic folding-frame wheelchair, essentially an indoor, low active-use chair, does not last under high active outdoors use:

‘I haven’t had this one long, but it is very feeble … They give in easily. This one is broken at the back and here too; [*shows the arm rest*] … Often the things get loose and get worked out … I had to swap one of these [*the footrest*] … [*because it*] … does not work.’ (P2, User, male, 63 years)

## Discussion

### Interaction between service steps

From the findings, it seems as if many of the service steps for wheelchair service delivery in the study setting were either not followed or were followed only superficially. There were implementation gaps with regard to the WHO guidelines (WHO 2008) and SA National guidelines (DoH 2003). This had a negative impact on wheelchair service delivery and, consequently, on user function. It is important to point out that service steps are interdependent. A challenge at any one point will affect negatively any preceding and subsequent steps as well as user outcomes.

This interdependence and its impact on user function are further explored through the example of prescription (service step 3). Findings indicated that prescription was not carried out according to policy. This can have a ripple effect on funding and ordering, fitting, training, maintenance and repairs. At the same time prescription cannot be divorced from assessment and referral and it is also influenced by service management and provider training.

If only one type of wheelchair is provided, as was the case in the study setting, it might be deemed unnecessary to perform prescription according to policy. It seems pointless to give the user a choice between different wheelchairs if only one type will be funded. Even so, limited choice is better than no choice and the wheelchairs provided in the study setting allow for choices such as type of upholstery, frame coating and colouring as well as wheel- and castor width. Users were not, however, afforded even these basic choices. In addition, showing users all types of wheelchairs can raise awareness amongst users. This in turn can lead to user advocacy in order to ensure their right to an appropriate assistive device. However, to implement this change with success it is necessary that service providers at both the district and clinic level receive training and that examples of the different options be made available for them for use during assessment and prescription. Whilst having a full range of all wheelchairs at every clinic might not be feasible, it must be possible (with the assistance of manufacturers) to have some physical examples as well as brochures and DVDs at each of the hospitals where assessing therapists perform outreach services.

Wheelchair assessment and prescription is a complex process, with many variables needing to be taken into account (Di Marco *et al*. 2003). The assessment should provide adequate information on the user’s lifestyle and social roles, level of functioning, environmental and postural support needs, cognitive and health needs, body measurements as well as safety and stability requirements to determine the specifications of an optimal wheelchair for the user (Greer *et al*. 2012; Moody *et al*. 2012; Whitcombe-Shingler 2004; WHO 2008). With the information gathered during wheelchair assessment in the study setting it would be difficult to prescribe an appropriate and safe wheelchair that will optimise user function and decrease the risk for accidents and secondary complications (Hansen *et al*. 2004; Moody *et al*. 2012).

Assessment and prescription are dependent on knowledgeable providers. Literature (DoH 2003; Greer *et al*. 2012; WHO 2008) emphasises that only appropriately-trained staff should provide wheelchair services. New graduates and newly-trained service providers require mentoring and guidance to deliver a comprehensive wheelchair service. The results of the study demonstrated a lack of knowledge and training of service providers in wheelchair provision as well as the absence of mentoring and support. Training packages are available in South Africa (Provincial Government of the Western Cape DoH 2009a, 2009b, 2010) and managers in the Northern Cape might be well advised to make sure that service providers receive this training.

However, a thorough assessment and correct prescription by adequately-trained providers will be of little value if the prescribed wheelchair is not funded or if the prescription is changed during the ordering process. Purchasing of wheelchairs in the public sector in South Africa is governed by a national tender document (South African National Treasury 2010). This document includes a comprehensive range of wheelchair options including manual, motorised or attendant-propelled and if appropriate for use in urban, rural and peri-urban settings. However, it seems as if financial constraints made only one type of wheelchair available to users in Fraserburg. The design characteristics of this chair (i.e. a relatively high seat, short wheelbase and no adjustability to optimise mobility and stability) make it unstable when used on uneven terrain. Unstable wheelchairs were linked to 16 fatal incidents in the UK over a three year period (Moody *et al*. 2012).

According to the procurement officer, the main challenge with regard to wheelchair service delivery was insufficient funds. A short term solution, namely, using funds earmarked for hearing devices, was used in order to address the wheelchair backlog. This impacts negatively on the rights of users with hearing impairments and does not provide sustainable funds for wheelchairs. This lack of funds might have been the cause of non-governmental organisations (NGOs) trying to relieve the need in any way possible as is described in the findings. Whereas such efforts may relieve some of the immediate need for wheelchairs, these *ad hoc* solutions are not sustainable and raise concerns about the suitability and appropriateness of the wheelchair (Mukherjee & Samanta 2005; WHO 2008). Literature indicates that unsuitable donated wheelchairs have a negative impact on user function, can cause injury and secondary complications and are often rejected (Mukerjee & Samanta 2005).

Thus funding, assessment, ordering and provider training can affect prescription negatively, either singly or in combination, and create a situation where an inappropriate wheelchair is prescribed. This in turn is sure to affect product preparation, fitting, user training and function. In a low-resource setting where wheelchairs may not fully match the prescription, product preparation is an essential step which allows modification and adaptation of the wheelchair to optimise the user’s function, health and postural support (WHO 2008). This further highlights the need for specific provider training, because in order to prepare the wheelchair optimally providers must be trained in a suitable manner.

Chaves *et al*. (2004) reported that 41% of wheelchair problems can be traced to poor fit. Fitting, carried out by trained providers, is critical in order to ensure postural stability and to prevent complications (DoH 2003; Hansen *et al*. 2004; WHO 2008). Optimal fitting is dependent on a comprehensive physical assessment of the user, correct prescription and optimal product preparation (Moody *et al*. 2012). If the wheelchair specifications do not adhere to the height, width, length and postural support requirements of the user, it might be impossible or at least very difficult to ensure optimal fitting.

Fitting in turn impacts user training. If the wheelchair does not provide proper postural support the user might sit in a position that is ergonomically suboptimal for self propulsion, operating safety devices such as the brakes, balancing on the back wheels and transferring. Appropriate training leads to significant improvement in general wheelchair mobility, increased mechanical efficiency and safety (De Groot *et al*. 2008; MacPhee *et al*. 2004; Ozturk & Dokuztug 2011) and increases the lifespan of the wheelchair (McAdam & Casteleijn 2005b). Users should understand the purpose and importance of every component of the wheelchair in order to ensure the safe use of their wheelchair (Whitcombe-Shingler 2004).

When an untrained user makes critical adjustments, such as changing the footrest height, it can have serious implications. Footrest height has a direct impact on postural stability and alignment, which may in turn have an impact regarding the risk for pressure ulcers, spasticity, contractures, poor organ function and decreased mobility (Hansen *et al*. 2004). No evidence could be found that users were taught pressure-relief techniques and methods for preventing contractures of the spine, pelvis and lower limbs – all of which are complications associated frequently with wheelchair use (Krause *et al*. 2008).

Another aspect that will affect training and user function is the suitability of the wheelchair to the environment. South African literature indicates that wheelchairs are ‘shelved’ if they are not suitable for the terrain in which users live and cannot be self-propelled on footpaths (Chakwiriza *et al*. 2010). Areas in the study setting are connected by a series of gravel roads and single-track footpaths. The wheelchair provided was not suitable for use on these roads (McAdam & Casteleijn 2005b).

The combined challenges in the service steps above may have a negative impact on maintenance and repair. Although the basic folding-frame wheelchair is cheaper than other models of wheelchairs, it is designed for low active, indoor or temporary (McAdam & Casteleijn 2005a) use and, therefore, when used in rural conditions, requires very high levels of maintenance and has an extremely short lifespan (Mukerjee & Samanta 2005). In addition, literature tells us that inappropriate wheelchairs are often abandoned or used less frequently which, in turn, impacts negatively on independence and quality of life (Mukherjee & Samanta 2005; Scherer *et al*. 2005). All this has negative financial implications for both users and the service provider (McAdam & Casteleijn 2005a). In addition, the repairs were performed far from the home setting, leaving users without mobility for the time taken to transport and repair the wheelchair. If they went with, they had to deal with traveling 800 km, the strangeness of a different city and city hospital and poor accommodation. Neither the cost, not the loss of independence created by this situation, is acceptable. Repair services should be contracted to a local supplier.

Thus challenges with regard to individual service steps as well as a combination of all the steps, result in poor outcomes which prevent users from reaching optimum levels of mobility and function and can cause complications and injuries (Chaves *et al*. 2004; Fogelberg *et al*. 2009; Mukherjee & Samanta 2005; Tomlinson 2000), resulting in an infringement of their basic rights. This is illustrated in the following narrative: ‘… it does not fit on the footpaths. You have to go on the road’ (P2, User, male, 63 years). The footpath was the shortest route by which this man with asthma and no transport could reach the CHCC when he required oxygen whilst having an asthma attack.

### The major factors limiting Policy Implementation and a Rights-Based Approach

The South African National Department of Health requires a rights-based approach to the provision of wheelchairs through the SA Guidelines (DoH 2003) and the NRP (DoH 2000). However, implementation of policy and guidelines is left to individual provinces and districts (DoH 2003). South Africa’s health system faces an array of challenges that hinder policy implementation, many of which are more pronounced in rural areas.

One of these relates to service providers. Literature indicates a serious shortage of service providers in rural areas (Cooke, Couper & Versteeg 2011). In addition, staff in rural areas often lack experience and specialised skills (Gaede & Versteeg 2011) and high staff turnover causes a lack of continuity (Van Deventer *et al*. 2008). Management is another area of healthcare provision in South Africa that experiences challenges (Mayosi *et al*. 2012; Naledi, Barron & Schneider 2011). Naledi *et al*. (2011) see the practice of controlling financial resources and decisions from provincial level as being one of the reasons for which district-level management fails. This practice creates role uncertainty and does not give district managers the autonomy to develop solutions to the challenges they experience.

This study identified many of the abovementioned staff-related and managerial challenges. In addition, wheelchair service delivery was challenged by the sheer size and remoteness of the GSA. The decision to manage wheelchair delivery services from the provincial level might have seemed reasonable in the light of the junior level of staff at the district and clinic level, as well as the high staff turnover. However, this practice limits access to wheelchairs for assessment and prescription and delays provision and fitting of the wheelchair. It seems an unsuitable choice in a province where distances are huge and poor road infrastructure creates challenges and is against Primary Health Care policy.

In addition, provincial mangers might lack local knowledge. For instance, managers at provincial level who are unfamiliar with the lack of road infrastructure in the study setting might have thought it appropriate to disregard policy and save money through providing only one type of wheelchair. However, managers at district level have better knowledge of local requirements and are thus in a better position to make decisions on the types of wheelchairs to issue.

Budgetary challenges impacted negatively on the ability of service providers to provide a service in accordance with policy and guidelines. All programmes for provision of assistive technology require dedicated business plans with clear objectives, outputs and outcomes. Business plans should include strategies to manage budgetary shortfalls and should consider options such as public or private partnerships and coordination of donations. However, the development of business plans requires adequately-skilled managers.

## Ethical considerations

The study received ethical clearance from the Committee for Human Research of the University of Stellenbosch. Permission to access the Fraserburg Community Health Care Centre (CHCC) and GSA was obtained from the Northern Cape Department of Health. Participation in the study was voluntary, confirmed by individual informed consent and did not influence adversely any future access to healthcare or wheelchairs on the part of the participants. All data gathered in the study were treated as confidential.

## Trustworthiness

Trustworthiness of data was achieved through prolonged engagement, triangulation, detailed description of the methodology, reflection and provision of a chain of evidence. Engagement with study participants occurred for various periods of time between 2010 and 2012. In addition, the first author was a member of the community and interacted with the community on a daily basis. Depth of data and understanding was sought through probing questions, engaging all senses during data collection, keeping field notes of observations made and going back to participants to further explore issues that were found to be unclear during provisional data analysis. Information from various data sources, such as service providers, service users and observations made by the researcher were triangulated. Interpretations and conclusions made in this article can be traced back to the results and discussion section. The information on which results and the discussion are based can in turn be found in the database, which is available for independent analysis or review. The authors tried to provide a clear description of the study methodology. This should provide readers with background should they wish to determine to what extent conclusions and recommendations can be transferred to other settings (Cohen & Crabtree 2006).

## Limitations

The findings presented in this paper are limited to one setting and qualitative study methods were used, thus whilst the authors’ clinical and research experience indicate that the setting has many similarities with other rural and remote settings in South Africa, extrapolation of findings and recommendations to other settings must be performed with caution.

Data were analysed by the primary author, without the use of computer software. Greater rigour might have been achieved if data were analysed and coded by more than one person or if a software programme were used.

## Recommendations

To improve users’ access to wheelchairs, wheelchair services providers and community sources should be re-orientated to recognise the wheelchair as a tool to realise basic human rights, such as a means to access health care services, provide dignity, alleviate the burden of care and to allow equal access to opportunities for education, employment, and community and social life (DoH 2003; Greer *et al*. 2012; UN 2006b; WHO 2008). In addition, to improve service delivery and access to services a comprehensive decentralised wheelchair service management programme, inclusive of all of the service steps and standards in accordance with the UNCRPD, WHO Guidelines and SA National Guidelines (DoH 2003; UN 2006b; WHO 2008) must be developed.

## Conclusion

This study provides preliminary evidence from one rural setting. Further exploration of wheelchair provision in other less-resourced settings as well as deductive studies to quantify challenges (Lang *et al*. 2011) is required to provide a comprehensive picture. However, the findings of this study showed important gaps between both local and international policy and service delivery. None of the eight steps of wheelchair service delivery according to the WHO guidelines were implemented in full (WHO 2008), nor were the SA guidelines (DoH 2003) or the UNCRPD implemented during wheelchair provision in the study setting.
